# Circulating Stromal Cell-Derived Factor 1α Levels in Heart Failure: A Matter of Proper Sampling

**DOI:** 10.1371/journal.pone.0141408

**Published:** 2015-11-06

**Authors:** Lesley Baerts, Yannick Waumans, Inger Brandt, Wolfgang Jungraithmayr, Pieter Van der Veken, Marc Vanderheyden, Ingrid De Meester

**Affiliations:** 1 Laboratory of Medical Biochemistry, University of Antwerp, Antwerp, Belgium; 2 Laboratory of Clinical Chemistry, OLV Hospital Aalst, Aalst, Belgium; 3 Division of Thoracic Surgery, University Hospital Zurich, Zürich, Switzerland; 4 Laboratory of Medicinal Chemistry, University of Antwerp, Antwerp, Belgium; 5 Cardiovascular Center, OLV Hospital Aalst, Aalst, Belgium; Indiana University School of Medicine, UNITED STATES

## Abstract

**Background:**

The chemokine Stromal cell-derived factor 1α (SDF1α, CXCL12) is currently under investigation as a biomarker for various cardiac diseases. The correct interpretation of SDF1α levels is complicated by the occurrence of truncated forms that possess an altered biological activity.

**Methodology:**

We studied the immunoreactivities of SDF1α forms and evaluated the effect of adding a DPP4 inhibitor in sampling tubes on measured SDF1α levels. Using optimized sampling, we measured DPP4 activity and SDF1α levels in patients with varying degrees of heart failure.

**Results:**

The immunoreactivities of SDF1α and its degradation products were determined with three immunoassays. A one hour incubation of SDF1α with DPP4 at 37°C resulted in 2/3 loss of immunoreactivity in each of the assays. Incubation with serum gave a similar result. Using appropriate sampling, SDF1α levels were found to be significantly higher in those heart failure patients with a severe loss of left ventricular function. DPP4 activity in serum was not altered in the heart failure population. However, the DPP4 activity was found to be significantly decreased in patients with high SDF1α levels

**Conclusions:**

We propose that all samples for SDF1α analysis should be collected in the presence of at least a DPP4 inhibitor. In doing so, we found higher SDF1α levels in subgroups of patients with heart failure. Our work supports the need for further research on the clinical relevance of SDF1α levels in cardiac disease.

## Introduction

In recent years, the chemokine Stromal cell-Derived Factor 1α (SDF1α or CXCL12) has been shown to play a role in cardiovascular diseases [1] and to be a promising biomarker [2,3]. Together with its receptor, CXCR4, SDF1α is involved in the homing of progenitor/stem cells thereby favoring the repair of injured myocardium through angiogenesis [1,4–6]. In addition, there is a growing interest in SDF1α as a cardiovascular biomarker. Elevated levels are associated with a risk of heart failure [2], the extent of coronary artery disease [3], and right ventricular dysfunction in patients with idiopathic pulmonary hypertension [7].

Similar to other chemokines, an intact N-terminus is essential for its biological activity [8]. Work by Crump et al. showed that loss of the N-terminal lysine, generating SDF1α_2–68_, results in a complete loss of bioactivity [9]. *In vivo*, N-terminal trimming is often initiated by dipeptidyl peptidase 4 (DPP4). Trimming by DPP4 results in the formation of SDF1α_3–68_ which not only lacks chemotactic properties but is also a powerful antagonist of the CXCR4 [9,10]. In this regard, Broxmeyer *et al*. showed that DPP4 inhibition significantly increases homing and engraftment of hematopoietic stem cells [11]. Other enzymes that might play a role in N-terminal cleavage are leukocyte elastase, matrix metalloproteases 1, 2, 3, 9, 13 and 14, and cathepsin G generating SDF1α_4–68_, SDF1α_5–68_ and SDF1α_6–68_ respectively. As mentioned, these cleavage products lack biological activity [12–14].

All these findings clearly demonstrate a crucial role for DPP4 and other proteases in modulating the biological activity of SDF1α. Moreover, DPP4 inhibitors or protease-resistant SDF1α analogs might become novel therapies in pathologies such as ischemic heart disease and heart failure [1,15–18]. In this case, the distinction between intact and cleaved SDF1α will become increasingly important to assess the biologically active SDF1α levels. Unfortunately, at present no commercially available immunoassay claims to discriminate between the intact and cleaved, and thus inactive, forms of SDF1α.

In this study, we first report on the difference in immunoreactivity between intact SDF1α and its cleavage products in commercial immunoassays. The addition of a DPP4 inhibitor to plasma tubes, as a means to prevent *ex vivo* proteolysis, profoundly affected the measurements. Secondly, the use of SDF1α and DPP4 as biomarkers were analyzed in patients with varying degrees of heart failure [19,20].

## Methods

### Enzymes and Inhibitors

Soluble human DPP4 was purified from seminal fluid as described previously [21]. One unit (U) of activity is described as the amount of enzyme required to catalyze the conversion of 1 μmol of substrate per minute (0.5 mM Gly-Pro-*p*-nitroanilide in 50-mM Tris buffer; pH 8.3) at 37°C. Diisopropyl fluorophosphate (DFP), an irreversible serine protease inhibitor, was purchased from Acros. Sitagliptin (SG), a specific DPP4 inhibitor (DPP4-I), was extracted from Januvia tablets (Merck). Vildagliptin (VG) another DPP4-I was custom-synthesized by GLSynthesis, Inc. Complete protease inhibitor cocktail tablets were purchased from Roche and used according to manufacturer’s instructions. These tablets inhibit a broad range of proteases including serine, cysteine as well as metalloproteases.

### DPP4 activities

DPP4 activities were measured using the fluorogenic substrate Gly-Pro-4-methyl-β-Naphtylamide as reported earlier [15]. In short, 10 μl sample was mixed with 100 μl of a 50-mM Tris buffer (pH 8.3) containing 0.5 mM Gly-Pro-4-methyl-β-Naphtylamide. The release of 4-methyl-β-Naphtylamide was measured for 10 min at 37°C (ʎ_ex_ = 340 nm; ʎ_em_ = 430 nm).

### ELISA and Antibodies

Two different lot numbers of CXCL12/SDF-1 DuoSet were purchased from RnDsystems (catalog N° DY460). The kit consists of a mouse anti-human/mouse SDF1α capture antibody, a biotinylated goat anti-human/mouse SDF1α detection antibody, and a Streptavidin-horseradish-peroxidase-(HRP) conjugate.

Experiments were repeated with two other ELISA kits: the human SDF1α mini ELISA Development Kit (Peprotech; catalog N° 900-M92) which consists of an anti-human SDF1α capture antibody, a biotinylated anti-human SDF1α detection antibody and an avidin-HRP conjugate and the human SDF1α ELISA Kit (Raybiotech; catalog N° ELH-SDF1a) which includes a plate precoated with capture antibody, a biotinylated anti-human SDF1α detection antibody, and a streptavidin-HRP conjugate.

The analyses were performed in Nunc Maxisorp 96-well plates for the RnD and Peprotech kit and in the supplied plate for the Raybiotech kit, according to manufacturer’s instructions. The readout was performed in a Tecan Infinite M200 microtiter plate reader.

### SDF1α Truncation

To study SDF1α truncation *in vitro*, the peptide provided by the CXCL12/SDF-1 RnD DuoSet was used for spiking (500 pg/ml). SDF1α was completely truncated through an incubation of one hour at 37°C in the presence of DPP4 [22]. As a control, DPP4 was first inhibited by a 10-min pre-incubation at 4°C with 1 mM DFP. To study *ex vivo* cleavage, serum (Bio-Rad; level 2 liquid assayed multiqual chemistry control serum) was spiked with SDF1α and incubated at room temperature (25°C) or 37°C for one hour. As a control, the serum was pre-incubated with protease inhibitors (100 μM SG and 1x complete protease inhibitor cocktail).

### Study population

Consecutive patients (age 65 ± 11 years) with a diagnosis of HfpeF (heart failure with preserved ejection fraction, > 40% and evidence of a left-ventricular dysfunction; n = 28) or HfrEF (heart failure with reduced ejection fraction, ≤ 40%; n = 30) [23] and a recent episode of decompensated heart failure, necessitating IV diuretic therapy, referred for diagnostic left and right heart catheterization were included in the study. Patients with HfrEF were further divided in those with compensated (characterized by a normal preload reserve) or decompensated heart failure (characterized by an impaired preload reserve) [24]. In addition, patients were categorized according to the ejection fraction in those with ‘normal’ ≥ 60%; ‘slight loss’ 59–51%; ‘loss’ 50–35%; ‘severe loss’ <35%. Patients with renal insufficiency defined by an estimated GFR (according to the modification of diet in renal disease study equation) below 60 ml/min/1.73 m^2^ or patients that received DPP4 inhibitors at the time of the study were excluded. All patients gave oral informed consent, a procedure which, at 2006, was approved by the local medical ethical committee of the OLV hospital, Aalst, Belgium. The study complied with the declaration of Helsinki. Patients were informed that the blood could be stored for the subsequent analysis of biomarkers. The oral informed consent was documented in the electronic or paper patient file and the study was approved by the local ethical committee.

Before diagnostic catheterization when the patient was in a stable hemodynamic condition five milliliter of whole blood was drawn from the femoral vein for subsequent measurements. Blood was collected in 7.5-ml EDTA tubes (S-monovette; Sarstedt) with or without DPP4-I to prevent *ex vivo* cleavage (VG, 120 μM final concentration in the tube). The samples were centrifugated for 15 min (2000 x g) and were subsequently frozen at –80°C until further analysis without undergoing additional freeze-thaw cycles. The plasma platelet number was not determined. Blood collected from patients with heart failure symptoms and without HfpeF or HfreF collected were chosen as control samples.

Catheterization of the left and right sides of the heart was performed unblinded from the right femoral artery and vein. Pulmonary capillary wedge pressure was measured by use of a Swan-Ganz catheter whereas LV pressure was recorded with a catheter, positioned in the left ventricular cavity. LV angiograms were obtained in left and right anterior oblique position. Left ventricular volumes and EF were derived from the single plane angiogram using the area-length method. An impaired preload reserve was defined by the presence of LVEDP ≥ 16 mm Hg [24].

### Statistics

Each measurement was performed 5 times and all measurements are reported as mean ± standard error of the mean (SEM). Specificity of the different ELISAs was compared using a Kruskall-Wallis analysis. When a significant difference was found, groups were compared with a Mann-Whitney-U test.

Cardiovascular parameters, SDF1α concentrations and DPP4 activities in the patient samples were analyzed using a one-way ANOVA followed by Bonferroni’s post-hoc tests if necessary.

All statistical analyses were performed by the *Statistical Package for the Social Sciences* (SPSS) version 20. Statistical differences were determined to be significant when the p-value was below 0.05.

## Results

### Specificity of SDF1α ELISAs

#### SDF1α and DPP4

**Fig 1:**
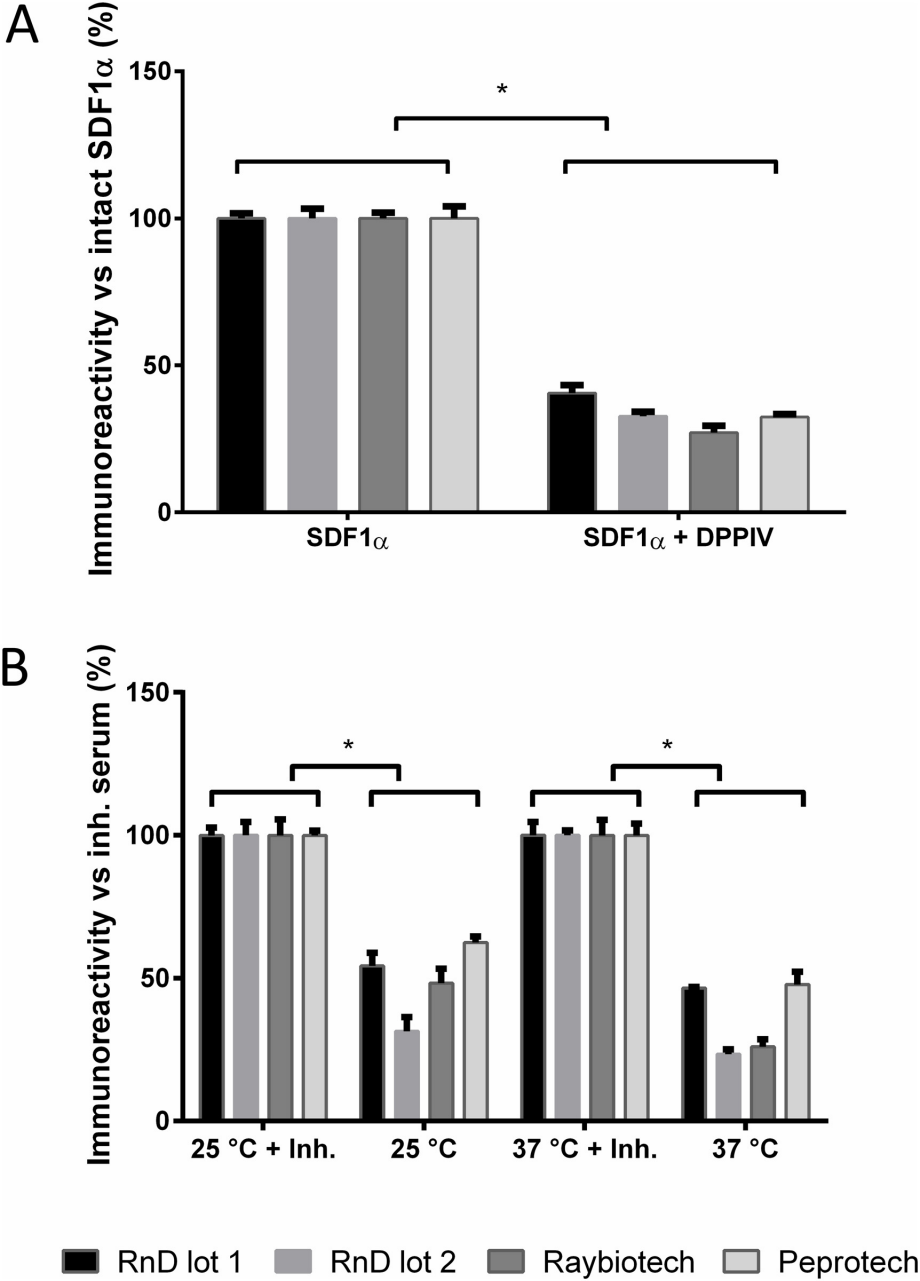
The SDF1α immunoreactivity measured by commercially available kits. (A) SDF1α (500pg/ml) incubated in PBS for 1 h at 37°C was set at 100% immunoreactivity. Incubation in the presence of DPP4 (25 U/l) resulted in a significantly lower immunoreactivity compared to intact SDF1α (RnD lot 1: 40.5 ± 2.8%; RnD lot 2: 32.7 ± 1.5%; Raybiotech: 27.1 ± 2.4%; Peprotech 32.5 ± 1.2%; *p < 0.05; results ± SEM; n = 5). (B) The immunoreactivity of pure SDF1α (500pg/ml) spiked into serum and incubated for 1 h at 25°C or 37°C was measured with different commercial kits. As the 100% reference, SDF1α spiked into inhibited serum (DPP4-I and Roche protease inhibitor cocktail) was chosen. *Ex vivo* degradation in serum significantly decreased the immunoreactivity of SDF1α (25°C: RnD lot 1: 54.3 ± 4.1%; RnD lot 2: 31.4 ± 6.4%; Raybiotech: 48.3 ± 5.1%; Peprotech: 62.4 ± 2.3% and 37°C: RnD lot 1: 46.6 ± 0.4%; RnD lot 2: 23.5 ± 1.7%; Raybiotech: 25.9 ± 2.7%; Peprotech 47.8 ± 1.9%; *p < 0.05; results ± SEM; n = 5).

The immunoreactivity of intact vs cleaved SDF1α was tested by incubating SDF1α with DPP4. After one hour at 37°C, a significantly lower immunoreactivity was detected for the DPP4-generated SDF1α_3–68_. This effect was observed with different lot numbers (RnDsystems) and different commercial ELISA kits (Raybiotech, Peprotech) (Fig 1A). As expected, the incubation of SDF1α_1–68_ with inactivated DPP4 did not result in a difference in immunoreactivity (S1 Fig).

#### SDF1α in serum

To study the *in vivo* cleavage of SDF1α by serum proteases including DPP4, serum was spiked with SDF1α and incubated at 25°C and 37°C for one hour. As a control, serum was inhibited beforehand with a combination of DPP4-I and protease inhibitor cocktail. Compared to control a significantly lower SDF1α immunoreactivity was found at 25°C and 37°C for all kits (Fig 1B). No differences in SDF1α immunoreactivity could be detected between the different kits and 25°C or 37°C.

### SDF1α in blood samples

#### Ex vivo cleavage of endogenous SDF1α

**Fig 2:**
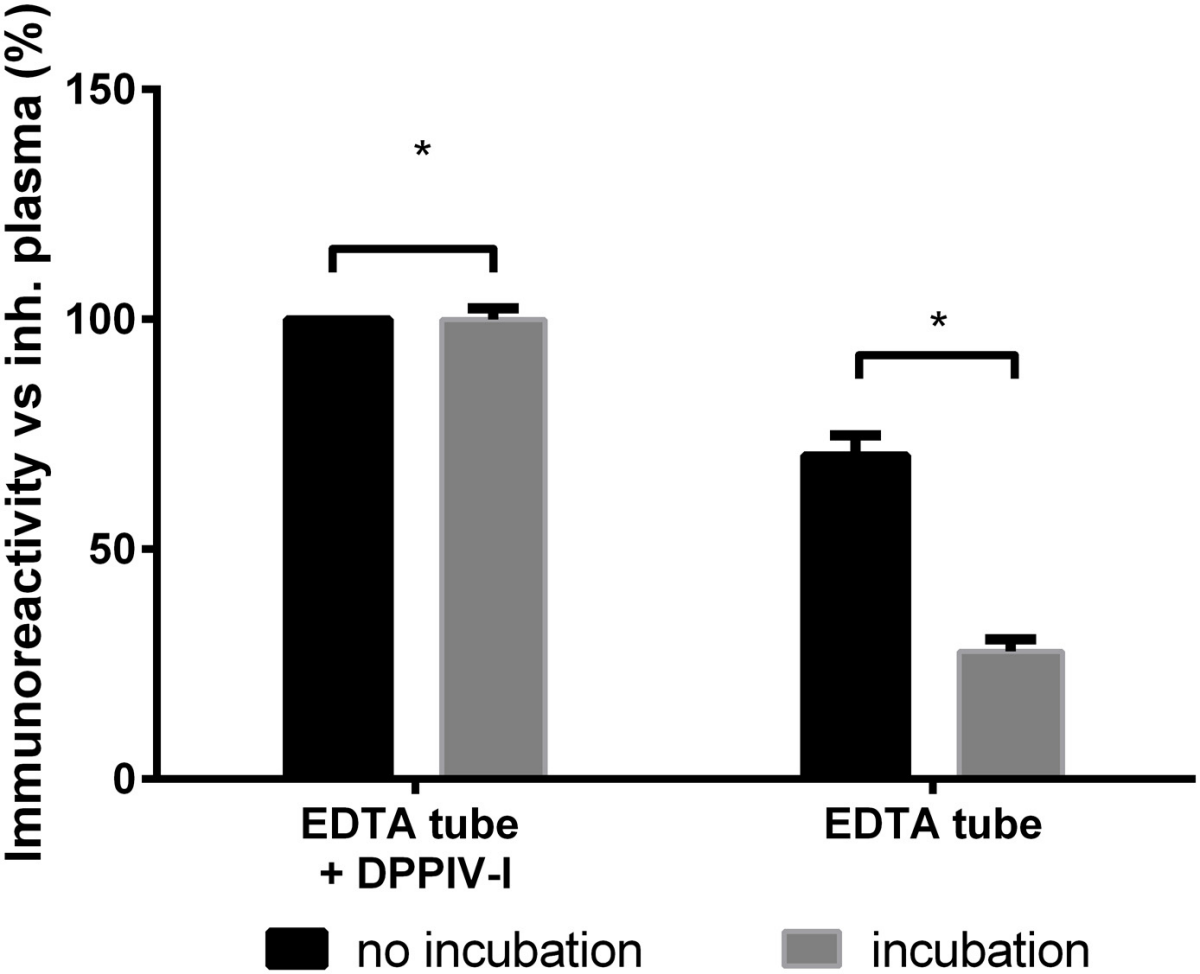
The average SDF1α immunoreactivity of healthy plasma with the RnD SDF1α duoset when immediately analyzed (no incubation, n = 13, range [749–1776 pg/ml]) or after an incubation of 1 h at 37°C (n = 7, range [832–1776 pg/ml]). Blood was collected in tubes with or without DPP4-I. A significantly lower immunoreactivity was found in regular tubes versus the DPP4-I containing tubes (no incubation: 67.6 ± 3.5%; incubation: 27.8 ± 2.8%; *p < 0.05; results ± SEM).

To examine the effect of *ex vivo* cleavage of endogenous SDF1α in plasma of healthy volunteers (n = 13). Blood was collected in EDTA tubes with or without DPP4-I, immediately processed and frozen at -80°C. The samples were thawed at 4°C and analyzed immediately. A significantly lower immunoreactivity was found in tubes that did not contain DPP4-I as compared to DPP4-inhibited samples (100%) (Fig 2).

Since samples are often not immediately analyzed, we also determined the immunoreactivity of endogenous SDF1α after an incubation of one hour at 37°C (n = 7). As expected, the immunoreactivity of the samples without DPP4 inhibitor was significantly lower compared to the DPP4-inhibited samples (Fig 2).

Finally, the immunoreactivity observed in DPP4-I samples did not rise further after addition of Roche Protease Inhibitor cocktail on top of the DPP4-I. (S2 Fig).

#### SDF1α levels, DPP4 activity and hemodynamic parameters

Characteristics of the study population are summarized in Table 1. In the entire study population, SDF1α levels ranged from 491 to 2550 pg/ml, median 1033 [915–1143] pg/ml.

**Table 1:** Patients characteristics according to Tertiles of SDF1α levels.

			SDF1α Tertiles		
		Tertile 1	Tertile 2	Tertile 3	p-value
		(n = 32)	(n = 32)	(n = 31)	
Mean SDF1α	pg/ml	836	1044	1369	
	*range*	*491–955*	*959–1119*	*1120–2550*	
Age	years	64	66	66	0.750
	*SD*	*8*	*12*	*10*	
Men	n	24	18	18	0.129
	*%*	*75*	*56*	*58*	
Heart Rate	bpm	73	67	72	0.386
	*SD*	*15*	*11*	*16*	
Ejection Fraction	%max	71	65	63	0.224
	*SD*	*18*	*17*	*20*	
EDP	mmHg	19	14	19	0.078
	*SD*	*8*	*4*	*16*	
EDVI	ml/m^2^	71	76	72	0.709
	*SD*	*26*	*27*	*19*	
ESVI	ml/m^2^	24	29	27	0.632
	*SD*	*21*	*21*	*21*	
DPP4 activity	U/l	24	23	20	**0.042**
	*SD*	*5*	*7*	*7*	
10-year survival	n	22	24	20	0.399
	*%*	*69*	*75*	*65*	

DPP4: Dipeptidyl Peptidase 4

SDF1α: Stromal cell-Derived Factor 1 alpha

**Fig 3:**
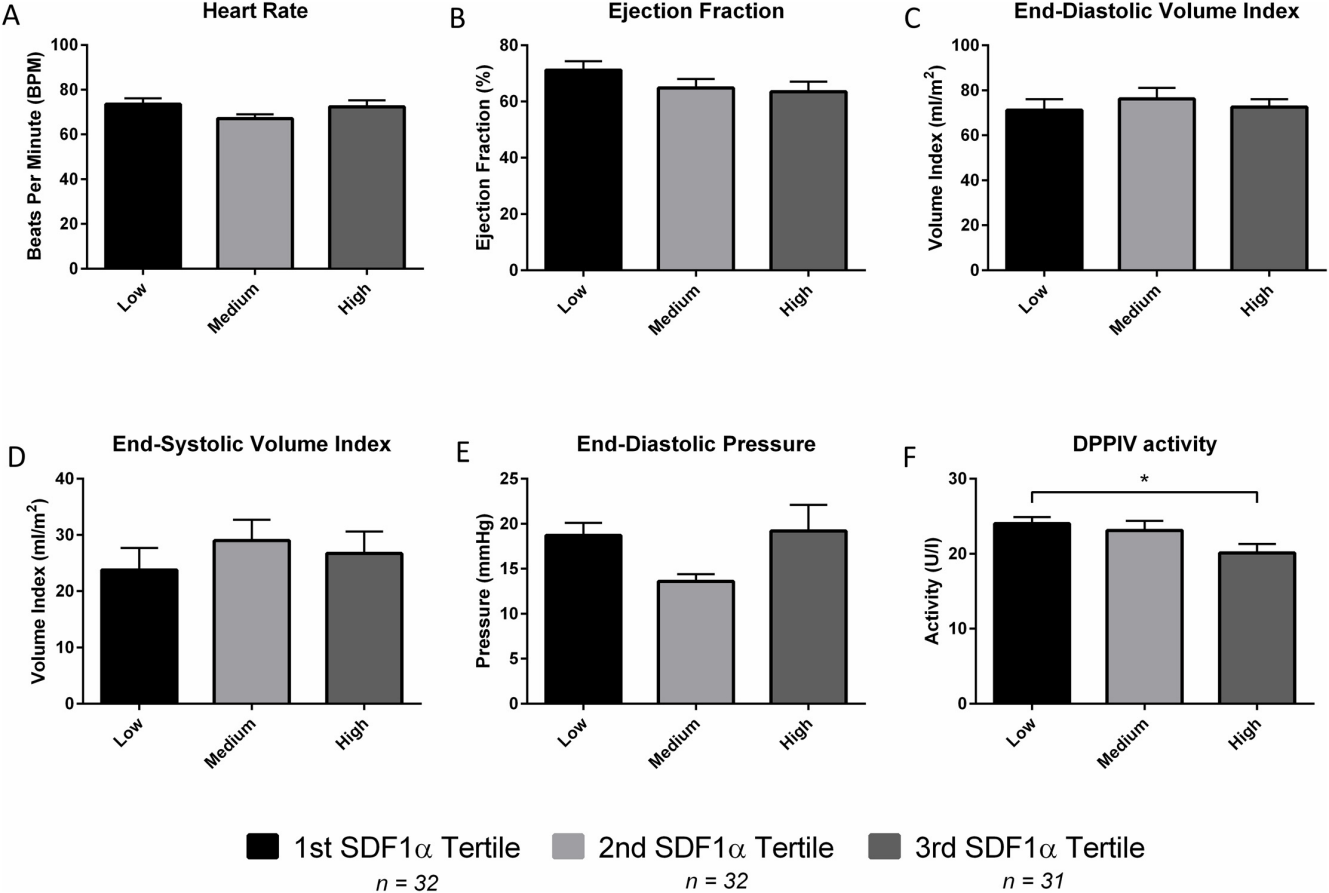
Comparison of cardiovascular parameters between SDF1α tertiles (Low = First Tertile, Medium = Second Tertile and High = Third Tertile). No significant differences were observed in the Heart Rate (A), Ejection Fraction (B), End-Diastolic Volume Index (C), End-Systolic Volume Index (D) and End-Diastolic Pressure (E). The first and third tertile had significantly different DPP4 activities (F). *p < 0.05.

Patients were divided into tertiles of SDF1α. A significantly lower DPP4 activity was found in patients with low compared to those with high SDF1α levels. Other cardiovascular parameters did not differ between groups (Fig 3).

**Fig 4:**
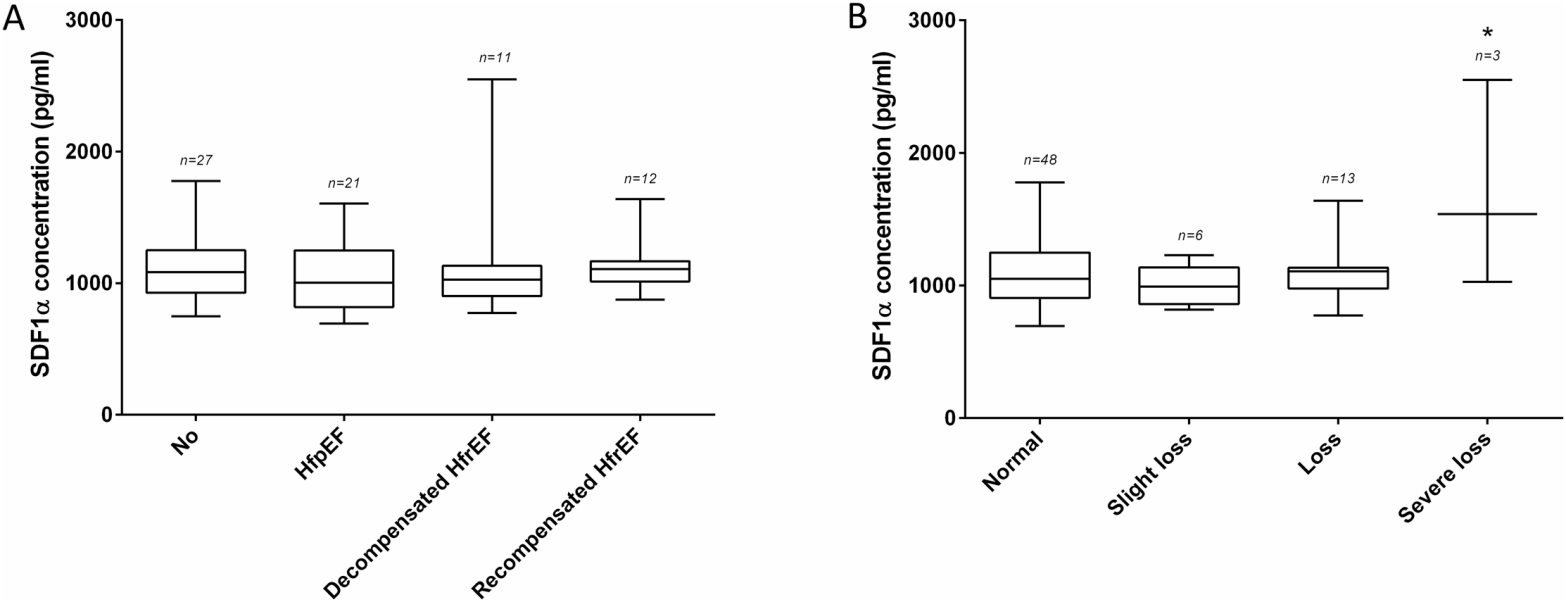
SDF1α concentrations in patient samples collected in tubes with DPP4-I. (A) No difference was found between patients with a different type of LV dysfunction (none 1096 ± 47 pg/ml; HfpEF 1043 ± 55 pg/ml; decompensated HfrEF 1201 ± 145 pg/ml; recompensated HfrEF 1109 ± 51 pg/ml). (B) For the different severities of LV dysfunction a significant difference was found in patients with a severe loss of LV function (normal 1076 ± 36 pg/ml; slight loss 1002 ± 62 pg/ml; loss 1090 ± 55 pg/ml; severe loss 1705 ± 447 pg/ml; *p < 0.05).

Although no difference in SDF1α levels was observed between controls, HfpEF and HfrEF patients (Fig 4A). Patients with severe LV dysfunction had significantly higher SDF1α concentrations (Fig 4B) DPP4 activity was similar in all of the investigated heart failure subgroups (S3 Fig).

## Discussion

### Quantification of the in vivo circulating SDF1α

SDF1α currently receives a lot of interest within cardiovascular research [2,5,18]. Although current clinical immunoassays have a high sensitivity and reproducibility, it remains unknown which kind of fragments these antibodies exactly recognize. As this leads to misinterpretation, a method to quantitate the *in vivo* circulating intact SDF1α was developed using commercially available immunoassays.

Our study revealed that frequently used commercially available immunoassays react differently towards intact SDF1α as compared to DPP4-truncated SDF1α. This might have clinical consequences as DPP4 circulates freely in the blood and is bound to the endothelial cell membrane. Consequently, DPP4 is able to lower the immunoreactivity both *in vivo* as well as *ex vivo* [25], resulting in an underestimation of physiological concentrations. In addition, other proteases might also contribute to SDF1α degradation [12–14]. However, the remaining immunoreactivity after incubation with DPP4 and serum was similar, which suggests that the observed *ex vivo* loss in signal is due to N-terminal truncation. The relative contribution of DPP4 was confirmed *ex vivo* with healthy plasma samples and is in line with previous findings of Kanki *et al*. [1]. This loss in immunoreactivity most likely reflects the immunogenic properties of the highly basic N-terminus. Even though we found this to be true for all tested immunoassays, these characteristics are often poorly specified by the manufacturer.

### Experimental and Clinical Implication

The *ex vivo* truncation, which occurs during sample handling, storage or even incubation of the ELISA plate, results in an underestimation of the physiological SDF1α concentrations. Of note, even under optimal pre-analytical conditions, there is a significant (more than 30%) loss of SDF1α- immunoreactivity in samples that did not contain a DPP4 inhibitor.

Our results show that *ex vivo* cleavage of SDF1α by DPP4 can lead to misinterpretations of experimental results. This is especially relevant when dealing with patients that are treated with DPP4 inhibitors or that have aberrant DPP4 activities, for example due to hyperglycemia or hypoxia [26, 27]. To overcome this problem, we highly recommend collecting all samples in tubes containing at least a DPP4 inhibitor to block any additional *ex vivo* splicing. The importance of this procedure is illustrated by the fact that an intact N-terminus is linked to SDF1α’s cardioprotective effects [1].

Our data is in line with other *in vivo* studies utilizing DPP4 inhibitors and measuring SDF1α levels. Most studies use the RnD immunoassay and all groups reported significantly higher SDF1α levels upon treatment with DPP4-I (S1 Table) [15,28,29]. Taking our results into account, these findings are suggestive of higher levels of intact and thus active circulating SDF1α.

The stabilization of *in vivo* intact SDF1α by DPP4 inhibition can be a valuable therapeutic strategy after myocardial infarction. Zaruba et al. showed that genetic deletion or pharmacological inhibition of DPP4 in combination with Granulocyte-Colony-Stimulating Factor led to improved heart function and survival [16]. Another strategy is to locally inject a protease-resistant SDF1α. This improves cardiac function after cardial ischemia and might provide an additional therapy for heart failure [1,30]. Clinical trials also indicate the potential use of SDF1α in cardiovascular pathologies. SDF1α gene therapy has been shown to be beneficial in a phase I study [18]. In its follow-up study (STOP-HF trial, NCT01643590), intra-myocardial delivery of SDF1α suggested a dose-dependent change in LVEF [31]. In the SITAGRAMI trial (NCT00650143), DPP4 inhibition in high doses was shown to increase the biological half-life of SDF1α and resulted in an improved cardial regeneration after myocardial infarction [32].

### DPP4 activity and SDF1α as biomarkers

In recent years, DPP4 activity and SDF1α have been suggested as possible biomarkers for heart failure [2,19,20]. Therefore, we evaluated both in patients referred for elective diagnostic cardiac catheterization.

We found DPP4 activities to be lower in patients with high SDF1α levels. It is of interest to note that the low DPP4 activities and high SDF1α levels might be related and that the difference in activity could point to the *in vivo* post-translational regulation of SDF1α [33]. In addition, patients with a severe loss of LV function showed a marked increase in SDF1α. Several mechanisms might be responsible for this observation. First, the higher wall stress with concomitant subendocardial ischemia may induce SDF1α production thereby mobilizing stem cells to the injured myocardium. Secondly, apart from beneficial effects, SDF1α might also have detrimental effects and depress LV function. It was recently shown that SDF1α has a negative inotropic effect through binding with its receptor CXCR4 [34]. Our results, in combination with a recent finding [2], point to the possible role of SDF1α as a biomarker in heart failure and warrant further investigation.

Surprisingly, no difference in DPP4 activities could be found between any of the investigated groups. This is in contrast with recent data demonstrating increased DPP4 activity levels in patients with heart failure [19,20]. One study focused on diastolic heart failure and only found a weak correlation with DPP4 activity in peripheral venous plasma [19], while a second study only included patients with a Left Ventricular Ejection Fraction lower than 45% [20]. As these studies had more stringent inclusion criteria for their heart failure patients, this could partially account for the observed discrepancy.

### Conclusion

We demonstrated that the N-terminal truncation of SDF1α profoundly affects the immunoreactivity measured by ELISA irrespective of the commercially available kit used. Additionally, we found that in the absence of protease inhibitors *ex vivo* cleavage cannot be prevented and that more than a third of the immunoreactivity is lost. We therefore recommend collecting all samples in tubes with protease inhibitors, at least including a DPP4 inhibitor. The possible value of DPP4 activities and SDF1α levels as biomarkers for heart failure was also evaluated. DPP4 activities were found to be lower in patients with high SDF1α levels. DPP4 activities were similar in the investigated heart failure subgroups. In contrast, SDF1α plasma levels were significantly elevated in patients with a severe loss of LV function. The observation of lower DPP4 activity in patients with high SDF1α as well as the presence of elevated SDF1α in patients with severe LV dysfunction warrants further investigation. Therefore, additional research is needed into the use of SDF1α as a biomarker and the role of DPPV as a target in patients with heart failure.

## Limitations of Our Study

The main limitation is the small number of severe heart failure patients included in our study. Therefore, data concerning this population should be interpreted with caution and confirmed with a larger population. A second limitation is the missing information regarding the epitopes recognized by the antibodies. Unfortunately, these could not be obtained from the suppliers as they considered it to be proprietary information. For the RnD duoset, we speculate that the mouse anti-SDF1 capture antibody is a monoclonal antibody that binds to an internal sequence. The goat anti-SDF1 antibody most probably is a polyclonal antibody that predominantly interacts with the N-terminus. As the polyclonal detection antibody is still able to interact with other parts of SDF1, the ELISA signal is strongly reduced, but not completely lost. The last limitation is that our proposed formula is based on a single time point and only gives a rough estimation of the circulating intact SDF1α.

## Supporting Information

S1 Fig Immunoreactivity of SDF1α after 1h incubation at 37°C with buffer, DPP4 or inhibited DPP4 (DPP4_inh;_ inhibited by a 10-min pre-incubation at 4°C with 1 mM DFP). SDF1α in buffer was selected as the 100% reference.
(PPTX)

S2 Fig Average immunoreactivity of endogenous SDF1α in healthy plasma after an incubation of 1 h at 37°C. (PPTX)

S3 Fig SDF1α concentrations in patient samples collected in tubes with DPP4-I. (A) No difference was found between patients with a different type of LV dysfunction (B) or for the different severities of LV dysfunction.
(PPTX)

S1 Table Overview of the articles utilizing DPP4 inhibitors and measuring SDF1α levels. (DOCX)

## Abbreviations

DPP4: Dipeptidyl Peptidase 4
DPP4-I: Dipeptidyl Peptidase 4 Inhibitor
ELISA: Enzyme-Linked ImmunoSorbent Assay
IH: Ischemic Heart Disease
LV function: Left Ventricular function
SDF1α: Stromal cell-Derived Factor 1 α
SG: Sitagliptin
VG: Vildagliptin
