# Supplementary figures and images for: Circulating Stromal Cell-Derived Factor 1α Levels in Heart Failure: A Matter of Proper Sampling
Source: PLoS One. 2015 Nov 6;10(11):e0141408. doi: 10.1371/journal.pone.0141408 (PMC4636157; doi:10.1371/journal.pone.0141408)

## Slide 1
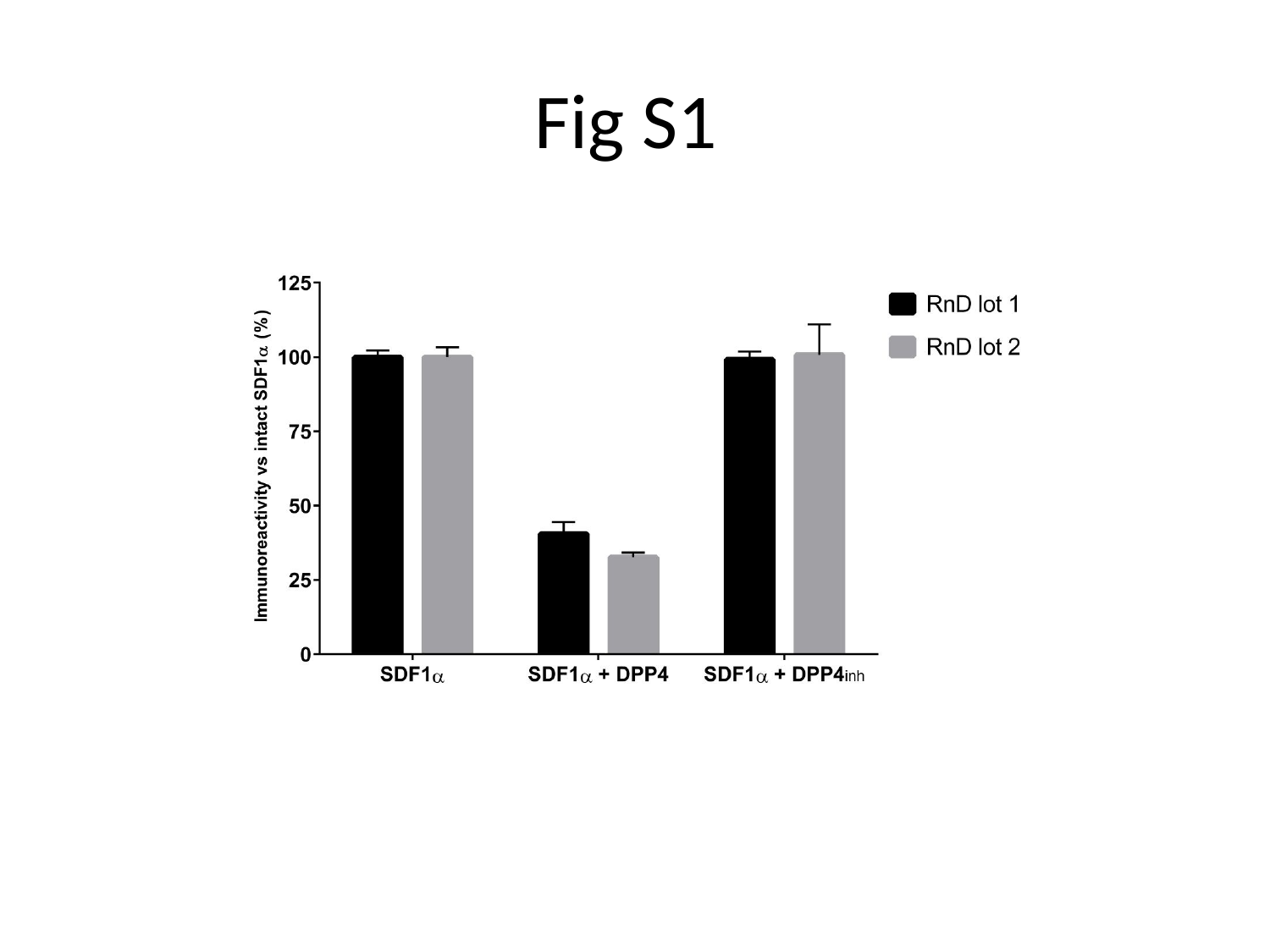

# Fig S1

Supplement: S1 Fig — SDF1α in buffer was selected as the 100% reference. (PPTX) [file pone.0141408.s001.pptx]

## Slide 1
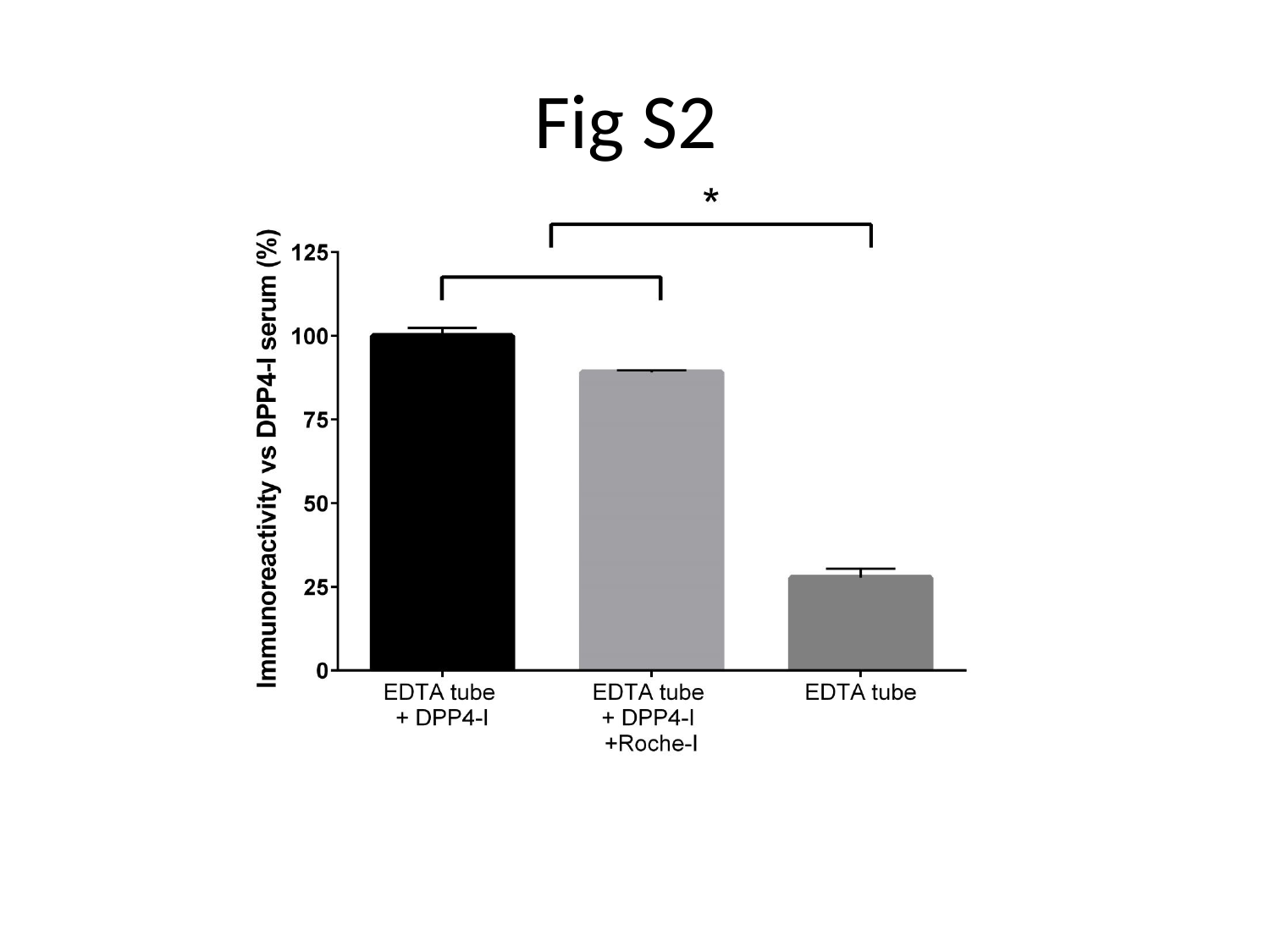

# Fig S2

Supplement: S2 Fig — (PPTX) [file pone.0141408.s002.pptx]

## Slide 1
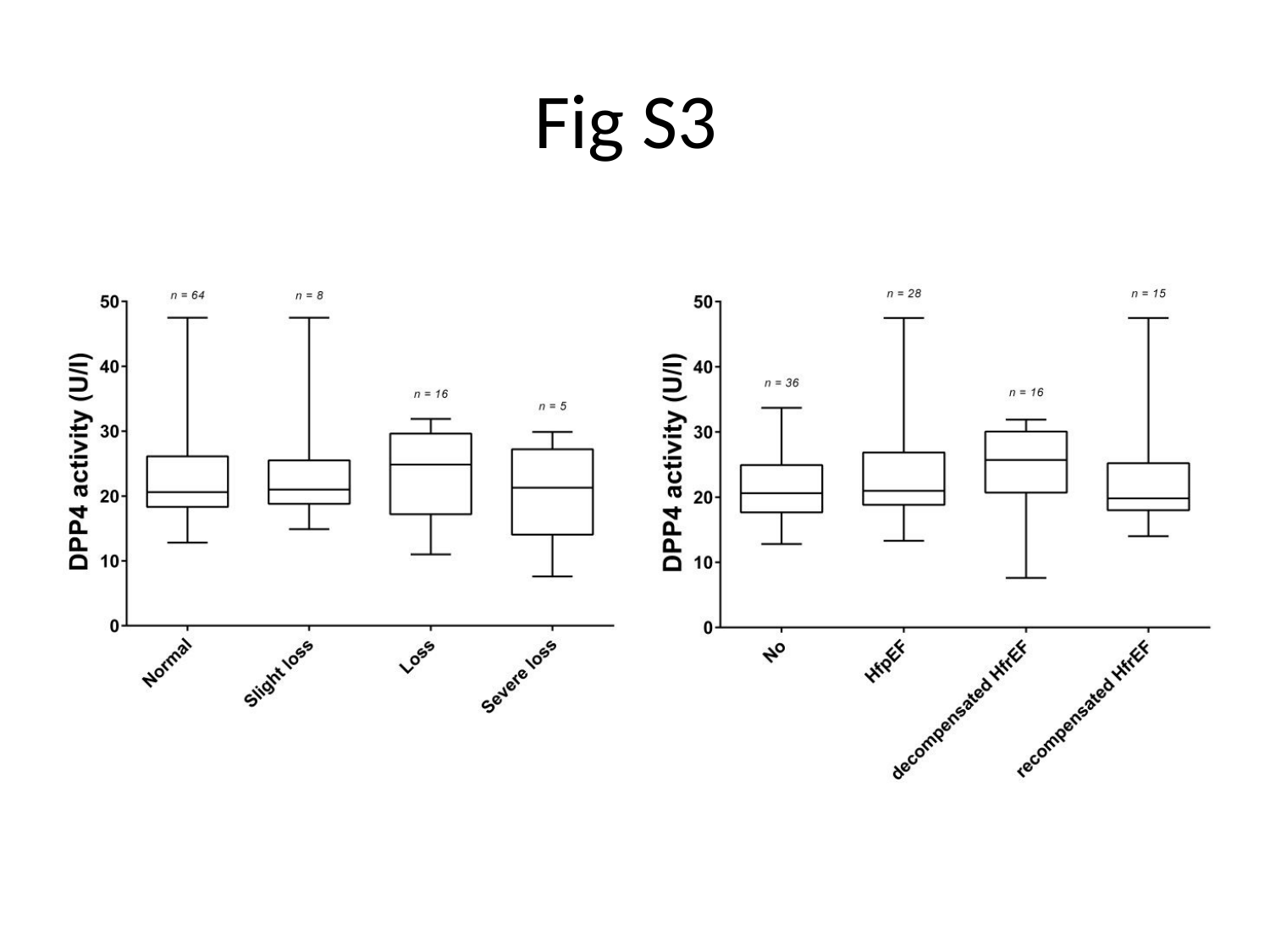

# Fig S3

Supplement: S3 Fig — (A) No difference was found between patients with a different type of LV dysfunction (B) or for the different severities of LV dysfunction. (PPTX) [file pone.0141408.s003.pptx]
