# Supplementary material for: Circulating Stromal Cell-Derived Factor 1α Levels in Heart Failure: A Matter of Proper Sampling
Source: PLoS One. 2015 Nov 6;10(11):e0141408. doi: 10.1371/journal.pone.0141408 (PMC4636157; doi:10.1371/journal.pone.0141408)
Supplement: S1 Table — (DOCX) [file pone.0141408.s004.docx]

**S1 Table: Overview of the articles utilizing DPP4 inhibitors and measuring SDF1α levels**

| **Authors** | **SDF Assay** | **Pathology** | **Setting/ Species** | **DPP4 Inhibitor treatment** | **Result** |
| --- | --- | --- | --- | --- | --- |
| Wang et al. 2006 [*] | RnD | EPC mobilization | murine BM | (indirect through enalapril)  1 mg/kg/day; s.c. 4 days before surgery | Enalapril increases plasma SDF1α and lowers BM SDF1α by respectively downregulating and upregulating DPP4 activity |
| Zaruba et al. 2009 [16] | RnD | ischemic cardiomyopathy | mice | Diprotin A  2 * 70 µg/kg/day ; i.p for 5 days | Genetic and pharmacologic inhibition of DPP4 does not significantly increase SDF1α myocardial levels |
| Ospelt et al. 2010 [**] | RnD | rheumatoid arthritis | mice and human synovial tissue | L-glutamyl L-boroproline  2 * 25 or 50 µg/day; oral for 60 days | DPP4 inhibition led to higher SDF1α levels |
| Fadini et al. 2010 [28] | multiplex array | EPC mobilization | diabetes type 2 patients | Sitagliptin  100 mg/day; oral for 4 weeks | 4 weeks of treatment with a DPP4 inhibitor led to higher plasma SDF1α levels |
| Huber et al. 2011 [†] | RnD | ischemic cardiomyopathy | mice | Parathyroid Hormone  80 µg/kg/day; i.p. for 6 days | PTH inhibits DPP4, which leads to increased cardial SDF1α levels |
| Jungraithmayr et al. 2012 [15] | RnD | lung transplantation | mice | Vildagliptin  Single dose 10 mg/kg; s.c. | Genetic and pharmacologic inhibition of DPP4 leads to higher SDF1α tissue levels |
| Huang et al. 2012 [29] | Raybiotech | EPC mobilization | mice | Sitagliptin  5, 10 or 20 mg/kg/day; oral gavage for 7 weeks | DPP4 inhibition leads to higher plasma SDF1 levels |
| Li , Wang 2013 [††] | RnD | Choroidal neovascularization, EPC mobilization | Murine plasma, BM | Imidapril  1 mg/kg/day; i.g.  5 days before and 14 days after surgery | Imidapril increases plasma SDF1α and lowers BM SDF1α by respectively downregulating and upregulating DPP4 activity |

Abbreviations: BM: Bone Marrow, EPC: Endothelial Progenitor Cell, i.g.: Intragastric, i.p.: Intraperitoneal, PTH: Parathyroid Hormone, s.c.: Subcutaneous

* Wang C-H, Verma S, Hsieh I-C, Chen Y-J, Kuo L-T, Yang N-I, et al. Enalapril increases ischemia-induced endothelial progenitor cell mobilization through manipulation of the CD26 system. J Mol Cell Cardiol. 2006;41: 34–43.

** Ospelt C, Mertens JC, Jüngel A, Brentano F, Maciejewska-Rodriguez H, Huber LC, et al. Inhibition of fibroblast activation protein and dipeptidylpeptidase 4 increases cartilage invasion by rheumatoid arthritis synovial fibroblasts. Arthritis Rheum. 2010;62: 1224–35.

† Huber BC, Brunner S, Segeth A, Nathan P, Fischer R, Zaruba MM, et al. Parathyroid hormone is a DPP-IV inhibitor and increases SDF-1-driven homing of CXCR4(+) stem cells into the ischaemic heart. Cardiovasc Res. 2011;90: 529–37.

†† Li H, Wang Y. An angiotensin-converting enzyme inhibitor modulates stromal-derived factor-1 through CD26/dipeptidyl peptidase IV to inhibit laser-induced choroidal neovascularization. Mol Vis. 2013;19: 1107–21.
